# Sensory Activation of Command Cells for Locomotion and Modulatory Mechanisms: Lessons from Lampreys

**DOI:** 10.3389/fncir.2016.00018

**Published:** 2016-03-22

**Authors:** Gheylen Daghfous, Warren W. Green, Simon T. Alford, Barbara S. Zielinski, Réjean Dubuc

**Affiliations:** ^1^Groupe de Recherche en Activité Physique Adaptée, Département des Sciences de l’Activité Physique, Université du Québec à MontréalMontréal, QC, Canada; ^2^Groupe de Recherche sur le Système Nerveux Central, Département de Neurosciences, Université de MontréalMontréal, QC, Canada; ^3^Department of Biological Sciences and Great Lakes Institute for Environmental Research, University of WindsorWindsor, ON, Canada; ^4^Laboratory of Integrative Neuroscience, Department of Biological Sciences, University of Illinois at ChicagoChicago, IL, USA

**Keywords:** sensorimotor, locomotion, modulation, reticulospinal neurons, lamprey, 5-HT

## Abstract

Sensorimotor transformation is one of the most fundamental and ubiquitous functions of the central nervous system (CNS). Although the general organization of the locomotor neural circuitry is relatively well understood, less is known about its activation by sensory inputs and its modulation. Utilizing the lamprey model, a detailed understanding of sensorimotor integration in vertebrates is emerging. In this article, we explore how the vertebrate CNS integrates sensory signals to generate motor behavior by examining the pathways and neural mechanisms involved in the transformation of cutaneous and olfactory inputs into motor output in the lamprey. We then review how 5-hydroxytryptamine (5-HT) acts on these systems by modulating both sensory inputs and motor output. A comprehensive review of this fundamental topic should provide a useful framework in the fields of motor control, sensorimotor integration and neuromodulation.

## Introduction

Locomotion is a rhythmic motor behavior involved in everyday functions. It requires the activation and coordination of the axial and/or appendicular musculature. Spinal neuronal networks called “central pattern generators” (CPGs) for locomotion generate the patterns of muscle activation that underlie propulsion during locomotion. Supraspinal structures, on the other hand, are required for activating and controlling the spinal CPGs. Descending inputs trigger, maintain, and eventually stop locomotion. The brainstem reticulospinal (RS) cells act as command cells that constitute an important interface between supraspinal and spinal networks. As such, the activation of RS cells by sensory (sensory-evoked locomotion) or internal clues (goal-directed locomotion) will markedly influence spinal function.

In this review, we focus on sensory-evoked locomotion by examining how two different sensory modalities influence the activation RS cells in a basal vertebrate, the lamprey. Because lampreys share a common brain “bauplan” with jawed vertebrates, including mammals, knowledge gained from neural circuits and mechanisms in lampreys provides insight into fundamental principles of vertebrate brain organization and function (Grillner et al., 1998b; Robertson et al., 2014). This review article focuses on some recent work in the lamprey from our labs on the pathways and neural mechanisms involved in the transformation of cutaneous and olfactory inputs into motor output. These sensory modalities are of paramount importance for the survival and reproductive success of individuals as they drive feeding, reproductive, and escape behaviors. We will also discuss 5-hydroxytryptamine (5-HT) modulation of these sensorimotor pathways. Indeed, 5-HT modulates both the sensory inputs to the RS cells at the supraspinal level and the descending motor commands of the RS cells in the spinal cord (SC). The mechanisms by which 5-HT modulates synaptic transmission has been well described in lampreys (Takahashi et al., 2001; Alford et al., 2003; Gerachshenko et al., 2005; Schwartz et al., 2005; Photowala et al., 2006; Schwartz et al., 2007; Gerachshenko et al., 2009; Alpert and Alford, 2013).

After a brief review of the motor circuitry and neural mechanisms of locomotion, sensorimotor transformations will be addressed starting with the neural pathways, from the receptors to the neural centers, followed by the neural mechanisms. Sensorimotor transformations of cutaneous and olfactory inputs will be addressed similarly. We have recently shown that the neural connections for these two sensory modalities differ considerably, yet activate the same target cells in the lower brainstem, the RS cells (Viana Di Prisco et al., 2000; Derjean et al., 2010). The pathway for cutaneous-induced locomotor reactions is shorter and more direct to the RS cells than the pathway involved in olfactory-induced locomotion. The transformation mechanisms are also different. The RS cell responses to cutaneous inputs (mechanical stimulation) switch from subthreshold excitatory postsynaptic potentials (EPSPs) to large sustained depolarizations; the output being an all-or-none escape locomotor bout. The olfactory inputs likely provide a more finely controlled locomotor output by acting via the mesencephalic locomotor region (MLR), a specific brainstem region involved in controlling goal directed locomotion. This review article will compare these two sensorimotor systems at the levels of neuronal connectivity and cellular mechanisms. We will then examine how 5-HT acts on these systems. In the past we have shown that cutaneous sensory inputs to RS cells are modulated by 5-HT (Antri et al., 2008). Similarly, there is prominent 5-HT innervation in the olfactory system, from the olfactory epithelium to the olfactory bulb (OB; Zielinski et al., 2000). Finally, we will review 5-HT modulation at the SC level. It has been extensively documented in the past that it exerts presynaptic effects that modulate the transmission from descending RS axons on SC neurons (Schwartz et al., 2005, 2007). This modulation has powerful effects on locomotor behavior.

## The Lamprey as a Vertebrate Model of Sensorimotor Integration

There is large interest in understanding the neural basis of behavior. The use of lampreys as a model system has made it possible to bridge the gap between cellular mechanisms and behavior. Indeed, the lamprey nervous system is remarkably similar to the mammalian nervous system, but it contains considerably fewer neurons and is thus greatly simpler. Moreover, lamprey brainstem command cells are more easily accessible for electrophysiological studies, which can be readily combined with imaging techniques in controlled *in vitro* conditions with the entire locomotor circuitry intact. The supraspinal mechanisms responsible for initiating and controlling locomotion can be studied with an array of *in vitro* techniques, with the added benefit of including all relevant structures needed for locomotor control, and the ability to monitor ongoing swimming behavior in a semi-intact preparation consisting of the exposed brain and rostral SC with the rest of the body left intact. As such, the lamprey model has paved the way for important discoveries, including the first detailed characterization of a vertebrate CPG for locomotion (Buchanan and Grillner, 1987). Acquisition of detailed knowledge of its motor circuitry opened the way for rapid progress in understanding sensorimotor integration at the system and cellular levels. For instance, new information on sensory-evoked locomotion was provided by describing the cellular mechanisms underlying the transformation of cutaneous inputs into locomotor output at the supraspinal level (Viana Di Prisco et al., 1997, 2000; Antri et al., 2009). For the first time in any vertebrate species, the neural substrate responsible for the transformation of olfactory inputs into a locomotor output was identified using the lamprey model (Derjean et al., 2010). Overall, the lamprey nervous system is ideally suited for the mechanistic study of sensorimotor integration.

## The Neural Control of Locomotion

As indicated above, the basic muscle synergies responsible for locomotor propulsion are generated by SC networks collectively known as CPGs (reviewed in Grillner et al., 2008). CPGs are also involved in generating respiration (reviewed in Del Negro et al., 2002) and mastication (reviewed in Westberg and Kolta, 2011). The neuronal activity is produced by integrating the intrinsic properties of the CPG neurons and the synaptic connectivity of the inextricably linked neural network (Marder and Thirumalai, 2002; Alford et al., 2003). Synaptic activity, whether mediating the release of fast acting neurotransmitters such as glutamate or neuromodulators such as the monoamines, dopamine or 5-HT, modifies the intrinsic properties. The neural network of the lamprey locomotor CPG has been well characterized (Buchanan and Grillner, 1987). Ipsilateral glutamatergic excitation in conjunction with contralateral inhibition play a crucial role (Grillner and Wallén, 1980; Buchanan and Cohen, 1982; Brodin et al., 1985; Alford and Williams, 1989; Hellgren et al., 1992); They generate ventral root (VR) bursting that alternates across the SC (Grillner et al., 1995). The spinal locomotor circuit is activated by descending commands and in particular by glutamate release from brainstem RS cells (Buchanan et al., 1987; Ohta and Grillner, 1989). The intensity of input from RS axons regulates the frequency of these bursts of activity and therefore the speed of locomotion (Viana Di Prisco et al., 2000; Brocard and Dubuc, 2003), which may range from 0.1 to 10 Hz. Experimentally, locomotor CPG activity in the SC may be activated by stimulating the lamprey brainstem in semi-intact preparations which generate RS output (McClellan and Grillner, 1984; Sirota et al., 2000; Brocard and Dubuc, 2003; Le Ray et al., 2003) or alternatively by applying glutamate receptor agonists in isolated SCs (Cohen and Wallén, 1980; Grillner et al., 1981). The alternating pattern of VR bursting recorded under these experimental conditions is referred to as “fictive locomotion”, and drives the coordinated contraction of muscles necessary for lamprey swimming.

The principal neurotransmitter that activates spinal CPGs is glutamate. Work in lampreys (Grillner et al., 1981; Buchanan et al., 1987; Marder, 1994), *Xenopus* tadpoles (Dale and Roberts, 1984; Roberts and Alford, 1986; Marder and Thirumalai, 2002; Alford et al., 2003), newborn rats (Armstrong, 1986; Kudo and Yamada, 1987) and cats (Shik et al., 1966; Douglas et al., 1993; Sirota et al., 2000) demonstrates that α-amino-3-hydroxy-5-methyl-4-isoxazolepropionic acid (AMPA) and N-methyl-D-aspartate (NMDA) receptor mediated transmission in the SC activates and maintains locomotion. These data are supported by direct recordings of EPSPs onto motoneurons and premotoneurons (Dale and Roberts, 1985; Brodin et al., 1988; Noga et al., 2003; Dubuc et al., 2008) and pharmacological manipulation of the resultant behaviors (Dale and Roberts, 1984; Brodin and Grillner, 1985; Cazalets et al., 1992; Chau et al., 2002; Rybak et al., 2006). Glutamatergic neurotransmission in the SC both directly excites neurons of the CPG, but may also activate either plateau properties of spinal cells as shown in the turtle (Hounsgaard and Kiehn, 1985; Guertin and Hounsgaard, 1998), or complex oscillatory properties in these neurons mediated by NMDA receptor voltage dependency and Ca^2+^ permeability. NMDA receptor-evoked neuronal oscillations were first shown in lamprey (Sigvardt et al., 1985; Wallén and Grillner, 1987), and were since identified in mammals (Hochman et al., 1994a,b; Wilson et al., 2005; Masino et al., 2012; for a review, see Schmidt et al., 1998).

**Figure 1:**
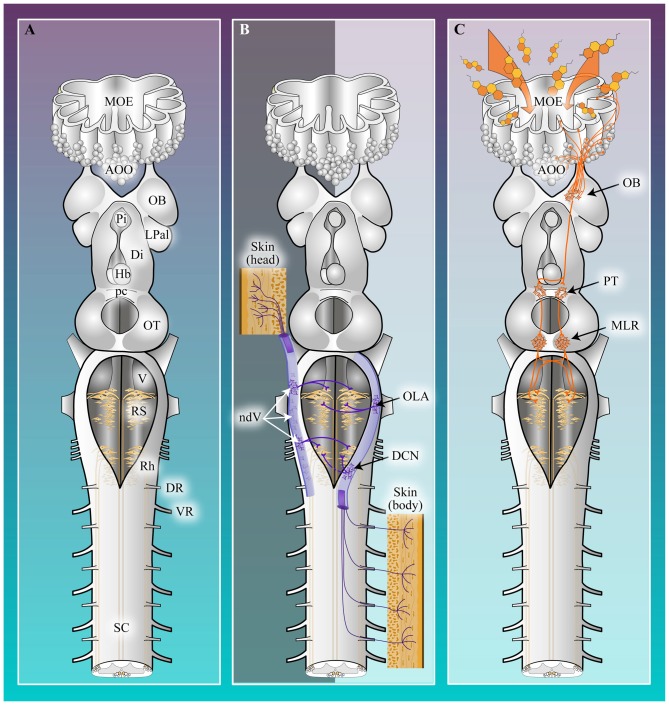
**Schematic representation of the brain and sensory-locomotor circuitry in lampreys. (A)** The lamprey central nervous system (CNS). AOO, Accessory olfactory organ; Di, Diencephalon; DR, Dorsal root; Hb, Habenula; LPal, Lateral pallium; MOE, Main olfactory epithelium; OB, Olfactory bulb; OT, Optic tectum; pc, Posterior commissure; Pi, Pineal gland; Rh, Rhombencephalon; RS, Reticulospinal cells; SC, Spinal cord; V, Motor nucleus of the trigeminal nerve; VR, Ventral root. **(B)** The somato-locomotor pathway (purple) involves only a single relay, located in the alar plate, between the afferent sensory fibers and the RS cells. The inputs from the head region are relayed to RS cells by neurons located in the nucleus of the descending root of the trigeminal nerve (ndV), whereas inputs from the body are relayed to RS cells by neurons located in the dorsal column nucleus (DCN) or in the octavolateralis area (OLA). **(C)** The olfacto-locomotor pathway (orange) consists of a projection from the medial part of the OB to the mesencephalic locomotor region (MLR) via the posterior tuberculum (PT). The MLR controls locomotion in all vertebrate species through a direct projection to the command cells for locomotion, the RS cells (beige). The RS cells, in turn, project to the spinal central pattern generators (CPGs) that generate muscle synergies responsible for locomotion.

The identity of the descending glutamatergic RS command neurons is well-defined in lampreys (Figure 1A; Dubuc et al., 2008). RS cells have been described anatomically and physiologically. They constitute about 90% of the neurones projecting to the SC (Swain et al., 1993; Bussières, 1994; Davis and McClellan, 1994a,b). RS cells are located in one mesencephalic reticular nucleus (MRN) and three rhombencephalic reticular nuclei, the anterior (ARRN), the middle (MRRN) and the posterior (PRRN; Nieuwenhuys, 1972, 1977; Brodin et al., 1988; Swain et al., 1993; Davis and McClellan, 1994a,b). There are about 1250 RS cells on each side and about 85% of these are located in the PRRN and MRRN (Bussières, 1994). Numerous attempts to establish homologies of these nuclei to reticular nuclei in other vertebrate species have been made in the past (Kimmel et al., 1982; Cruce and Newman, 1984; ten Donkelaar et al., 1987; Nieuwenhuys and Nicholson, 1998; Brocard and Dubuc, 2003; Butler and Hodos, 2005; Nieuwenhuys, 2011). Based on the cytoarchitecture, anatomical position and connections of the reticular nuclei, the following homologies were proposed: (i) the ARRN and MRRN, which contain large medially-projecting RS cells (Müller cells; Rovainen, 1967), are homologous to the superior and middle reticular nuclei of fish, amphibians and reptiles. These nuclei would be respectively homologous to the nuclei pontis oralis and caudalis of mammals; (ii) the PRRN, which contains laterally-projecting RS cells, is homologous to the inferior reticular nuclei of fish, amphibians and reptiles. This nucleus is comparable to the nuclei reticularis gigantocellularis, ventralis and magnocellularis of mammals.

It has been shown in lampreys that the axons of large RS cells make synaptic contacts with several classes of spinal neurons and some are involved in generating locomotion (Rovainen, 1974; Buchanan, 1982; Ohta and Grillner, 1989; for a review of RS pathways in mammals, see Perreault and Glover, 2013). Similarly, studies conducted in mice and zebrafish showed that genetically identified glutamatergic RS cells of the hindbrain are involved in controlling locomotion (Hägglund et al., 2010; Bretzner and Brownstone, 2013; Kimura et al., 2013). Bouvier et al. (2015) recently identified a new population of RS cells involved in stopping locomotion. These studies opened the way to the genetic dissection of RS pathways, thus leading to further examination of their evolutionary conservation. As will be outlined below, locomotor activity is produced by spinal CPGs that are activated and maintained by descending commands. During goal-directed locomotion, this activity originates in forebrain structures, including the basal ganglia (Armstrong, 1986), to activate a largely serial process in which these higher centers recruit locomotor centers including the MLR (Shik et al., 1966; Eidelberg et al., 1981; Skinner and Garcia-Rill, 1984; Bernau et al., 1991; Marlinsky and Voitenko, 1991; Sirota et al., 2000; Cabelguen et al., 2003; Musienko et al., 2008; Ryczko et al., 2016; for reviews, see Le Ray et al., 2011; Ryczko and Dubuc, 2013) and then the RS system (Orlovskiĭ, 1970a; Orlovskiĭ, 1970b; Orlovskiĭ, 1972; Shefchyk et al., 1984; Steeves and Jordan, 1984; Garcia-Rill et al., 1986; Jordan, 1986; Garcia-Rill and Skinner, 1987a,b). The MLR exerts an excitatory influence on RS cells either directly via glutamatergic and cholinergic (nicotinic) projections to the RS cells (Brocard and Dubuc, 2003; Le Ray et al., 2003; Noga et al., 2003; Grillner et al., 2008; Brocard et al., 2010) or indirectly via a cholinergic projection to glutamatergic muscarinoceptive cells of the brainstem that project back to the RS cells (Smetana et al., 2007, 2010). The involvement of the cholinergic system in the control of locomotion is still subject to debate. Indeed, MLR cholinergic inputs have an excitatory influence on RS cells, but are not essential to induce locomotion (Brocard and Dubuc, 2003; Le Ray et al., 2003; Smetana et al., 2007, 2010; Jordan et al., 2014; Roseberry et al., 2016). As such, it seems that cholinergic inputs cooperate with glutamatergic inputs to amplify and sustain the locomotor output. Further investigations of the respective roles of the glutamatergic and cholinergic systems in the control of locomotion are needed.

## Sensorimotor Transformations of Cutaneous Inputs

Cutaneous inputs can induce, modulate and stop locomotion in vertebrates (Duysens, 1977; Viala et al., 1978; Clarke and Roberts, 1984; McClellan and Grillner, 1984; Boothby and Roberts, 1992; Frigon et al., 2012; reviewed in Grillner, 1985; Rossignol et al., 2006). The anatomical pathways and cellular mechanisms underlying these sensorimotor transformations were first characterized in the lamprey (Dubuc et al., 1993a,b; Viana Di Prisco et al., 1997, 2000, 2005; Antri et al., 2009; Le Ray et al., 2010) and more recently in *Xenopus* (Buhl et al., 2012).

### The Neural Pathway: From Cutaneous Receptors to Brain Locomotor Centers

As in other vertebrates, different types of mechanoreceptors are located on the skin of lampreys (Lethbridge and Potter, 1982). These include Merkel cells (Fahrenholz, 1936; Whitear and Lane, 1981), free nerve endings, and neuromasts (Lethbridge and Potter, 1982). Neuromasts are mechanosensory organs associated with the lateral line system and will not be discussed further here (see Gelman et al., 2007). The Merkel cells are present all over the body and are particularly abundant in the epidermis of the mouth, gills and fins (Fahrenholz, 1936; Whitear and Lane, 1981; Lethbridge and Potter, 1982). As seen in other vertebrates, these microvillar cells are connected to the surrounding epidermal cells by desmosomes and granules are concentrated at the site of apparent synaptic junctions with afferent nerve fibers. However, in the lamprey, their association with a nerve fiber is unique by the presence of a spur on the neurite (Whitear and Lane, 1981). Nerve fibers conveying cutaneous input enter the CNS by the dorsal roots (DRs) of the SC (body inputs) and by the trigeminal nerve (head inputs) (Figure 1B; Martin and Wickelgren, 1971; Matthews and Wickelgren, 1978; Rovainen and Yan, 1985; Christenson et al., 1988). Afferent fibers of the DRs and trigeminal nerve have their cell bodies within the brain/SC (dorsal cells) or in the DR ganglia/trigeminal ganglia (Rovainen and Yan, 1985). Most DR afferent fibers carrying somatosensory information from the body ascend, via the dorsal column, to terminate in the dorsal column nucleus (DCN) in the brainstem (Figure 1B; Dubuc et al., 1993b). Some fibers, however, continue further rostrally and reach the octavolateralis area (OLA; Figure 1B; Ronan and Northcutt, 1990; Dubuc et al., 1993b). Alar plate neurons from the DCN and OLA then project to the RS cells thus providing a disynaptic pathway for cutaneous inputs to reach the RS cells (Figure 1B; Dubuc et al., 1993b; Pflieger and Dubuc, 2004). Similarly, there is a disynaptic pathway relaying head somatosensory information to the RS cells (Figure 1B). After entering the brain by the sensory root of the trigeminal nerve, trigeminal afferent fibers form the descending root of the trigeminal nerve (dV) that extends down to the rostral SC. There is no well-defined sensory nucleus of the descending root (ndV) in lampreys but neurons scattered among the dV fibers constitute a diffuse ndV (Northcutt, 1979; Viana Di Prisco et al., 2005). Interestingly, tract-tracing experiments showed that some of these neurons project to the RS cells and thus could constitute a trigeminal sensory relay to RS cells (Viana Di Prisco et al., 2005).

### The Neural Mechanisms

Cutaneous primary sensory neurons (ganglion and dorsal cells) have been classified as touch, pressure, and possibly nociception, based on their response patterns to the skin stimulation. Touch cells are fast-adapting cells that respond to light mechanical stimulation of the skin with one or two spikes at the onset and offset of the stimulation (Martin and Wickelgren, 1971; Matthews and Wickelgren, 1978; Christenson et al., 1988). Pressure cells respond to mechanical stimulation of the skin by a slowly-adapting discharge with frequency related to the stimulus intensity. A third type of cell, the nociceptive cell, has been reported to respond to heavy pressure applied on the skin by a slowly-adapting discharge (Martin and Wickelgren, 1971; Matthews and Wickelgren, 1978; Rovainen and Yan, 1985). Activation of these primary sensory neurons by mechanical stimulation of the skin or electrical stimulation of the nerves (trigeminal or spinal DRs) induces post-synaptic potentials in intracellularly recorded RS cells (Viana Di Prisco et al., 1997, 2000, 2005). This finding is in accordance with behavioral observations showing that skin stimulation elicits escape swimming in intact animals (McClellan, 1988; Cardin et al., 1999). Excitatory and inhibitory amino acids are involved in the transmission of cutaneous inputs to RS cells (Dubuc et al., 1993a,b; Viana Di Prisco et al., 1995, 2005). The transmission from cutaneous (trigeminal and dorsal column) sensory afferent fibers to alar plate relay neurons was shown to be glutamatergic (Dubuc et al., 1993a,b; Viana Di Prisco et al., 1995, 2005). Cutaneous inputs are then relayed to RS cells by glycinergic and glutamatergic neurons of the relay nuclei (OLA and DCN for body inputs; dV for head inputs).

Further examination of the physiology of this disynaptic pathway led to a very interesting discovery on how RS cells transform a brief sensory input into a long-lasting motor output due to intrinsic plateau properties of RS cells (Viana Di Prisco et al., 1997). Indeed, the skin stimulation intensity—RS cell response intensity relationship is not strictly linear. At low intensities, skin stimulation elicits graded post-synaptic potentials in RS cells in a linear fashion. As the sensory stimulus intensity reaches a high level, the excitatory response in RS cells switches from sub-threshold to a large sustained depolarization that triggers escape locomotion in a semi-intact preparation. The sustaining depolarization is NMDA receptor-dependent and Ca^2+^ entry into the cell in turn activates a Ca^2+^-activated non-selective cation current (I_CAN_). The sustained depolarizations often last for a very long duration (up to minutes). It was found that synaptic inputs could feed back onto intrinsic properties to temporally amplify the sustained depolarizations (Antri et al., 2009). Reversibly blocking SC inputs to RS cells markedly reduced the duration of the sustained depolarizations. In addition, pressure ejecting ionotropic glutamate receptor blockers on a recorded RS cell during the sustained depolarization reduced both their amplitude and duration. These findings indicate that excitatory synaptic inputs cooperate with intrinsic properties to prolong the sustained depolarizations (Antri et al., 2009). Whether similar mechanisms are involved in transforming sensory inputs from other sources into motor output remains to be determined.

## Sensorimotor Transformations of Olfactory Inputs

Olfactory cues can induce locomotion in vertebrates (Hasler and Wisby, 1951; Fady et al., 1998; Varendi and Porter, 2001; Johnson et al., 2009; for a review, see Daghfous et al., 2012). However, the neural substrate underlying olfactory-activated locomotion has long eluded characterization. Pioneering work (Grimm, 1960; Døving and Selset, 1980) showed that electrical stimulation of the olfactory tracts in fishes induced stereotyped motor behaviors including locomotion. Moreover, recent investigations of neural circuits and cellular mechanisms in the sea lamprey have unraveled how olfactory inputs can initiate locomotion (Derjean et al., 2010).

### The Neural Pathway: From Olfactory Sensory Neurons to Brain Locomotor Centers

In lampreys, a single nostril located along the midline on the dorsal surface of the head, anterior to the eyes opens into a single nasal cavity containing the peripheral olfactory organ. The walls of this cavity form folds or lamellae lined by an epithelium containing olfactory sensory neurons (OSNs). These lamellae house the main olfactory epithelium (MOE), and contain three ciliated OSN morphotypes: tall, intermediate and short OSNs (Laframboise et al., 2007). Their shapes and locations are similar to OSN morphotypes present in teleost fishes (Hansen and Zielinski, 2005). Diverticula (i.e., epithelial vesicles) of the MOE, mainly located in the caudoventral part of the olfactory organ, form the accessory olfactory organ (AOO) of lampreys (Scott, 1887; Leach, 1951; Hagelin and Johnels, 1955). The lumina of the AOO diverticula are linked to the lumen of the olfactory organ by tiny ducts (Hagelin and Johnels, 1955), and the cuboidal epithelium lining the AOO vesicles contains ciliated short OSNs with a broader surface than the short OSNs in the MOE (Ren et al., 2009; Chang et al., 2013). Axons extend from both MOE and AOO into the underlying lamina propria, where small axonal bundles gather to form the olfactory nerve. These OSN axons enter the OB, the primary olfactory center of the brain, where synaptic contacts are made onto the second order olfactory neurons, the OB projection neurons (the equivalent of the “mitral/tufted” cells of mammals). Axons from AOO OSNs terminate only in the medial part of the OB whereas axons from MOE OSNs terminate in non-medial parts of the OB and possibly sparsely in the medial part of the OB (Ren et al., 2009). Interestingly, OSN axons extending into medial and non-medial regions of the OB have distinct biochemical properties (Frontini et al., 2003). Moreover, projection neurons located in the medial and non-medial part of OB have non-overlapping receptive fields and exhibit differences in size and dendritic morphology (Green et al., 2013). The projection neurons send their projections to third order olfactory neurons in different parts of the brain. Structures receiving these secondary olfactory projections are located mainly in the telencephalon, but some secondary olfactory fibers extend to the mesodiencephalic boundary. Third order olfactory neurons are located in the septum, striatum, pallium, habenula, hypothalamus as well as the posterior tuberculum (PT), a ventrocaudal region of the diencephalon. Tract-tracing experiments revealed that the olfactory connection to the PT originates exclusively from the medial projection neuron population (Figure 1C), whereas connectivity to the other aforementioned areas arise from non-medial projection neurons. Conversely, the PT appears to be the only target of the projection neurons of the medial OB (Figure 1C; Figure 5 in Derjean et al., 2010; Green et al., 2013). This OB projection to the PT is of special interest as it was previously shown that the PT sends downward inputs to the MLR (Figure 1C; Ménard et al., 2007). The MLR is a crucial motor center located at the border between the mesencephalon and the pons. In all vertebrate species, it controls locomotion in a graded fashion, via projections to RS cells (for reviews, see Dubuc et al., 2008; Ryczko and Dubuc, 2013). Thus, the projection from the medial OB to the PT provides a way for olfactory inputs to influence locomotion in a very direct fashion (Figure 1C).

### The Neural Mechanisms

The activation of OSNs by chemical stimuli constitutes the first step of any olfactory-mediated behavior. In lampreys, OSNs have been shown to respond to three major classes of chemical stimuli: amino acids, steroids, and bile salts (Li et al., 1995). Stimulation of the olfactory epithelium with some of these naturally occurring olfactory stimuli can induce sustained depolarizations with spiking activity in RS cells. The stimulatory molecules include the sex pheromones 3-keto petromyzonol sulfate and 3-keto allocholic acid as well as odors such as taurocholic acid and L-arginine (Figure 1 in Derjean et al., 2010). Similarly, electrical stimulation of the olfactory nerve elicits excitatory synaptic responses in intracellularly recorded RS cells (Wickelgren, 1977; Brodin et al., 1988; Figure 2 in Derjean et al., 2010). Responses occur on both sides with a latency of around 100 ms. Calcium imaging experiments confirmed this finding by showing that repetitive stimulation of the olfactory nerve increases intracellular calcium in many RS cells (Figure 2 in Derjean et al., 2010), a sign of long-lasting afterdischarges in these cells (Viana Di Prisco et al., 1997). Local injections of glutamate antagonists in the OB blocked the responses of RS cells to olfactory nerve stimulation, indicating that synaptic transmission between OSNs and projection neurons relies on glutamate (Figure 2 in Derjean et al., 2010). The glutamatergic nature of this synapse was confirmed by showing that glutamate injection into the OB induces fictive locomotion (Figure 3 in Derjean et al., 2010). Examination of RS cell responses following the stimulation of different OB regions revealed that stimulating the medial region of the OB was more effective than stimulating non-medial regions (Figure 4 in Derjean et al., 2010). In summary, anatomical evidence and physiological experiments emphasise the role of the medial OB region in the fast relay of olfactory inputs to locomotor centers.

Because projection neurons from the medial OB region only project to the PT, the effect of PT stimulation on RS cell activity was investigated. Electrical stimulation of the PT elicited excitatory responses in RS cells with a latency of around 15 ms. Pharmacological stimulation of the PT with glutamate induced locomotor bouts in semi-intact preparations (Figure 6 in Derjean et al., 2010; Ryczko et al., 2013). Moreover, glutamate antagonist injections into the PT abolished RS cells responses to olfactory nerve stimulation, demonstrating that the OB projections to the PT are glutamatergic and that the PT is involved in olfacto-motor transformations (Figure 7 in Derjean et al., 2010). Because the MLR receives inputs from the PT (Ménard et al., 2007) and projects to RS cells (Sirota et al., 2000), it is an ideal candidate to relay PT olfactory inputs to RS cells. Physiological data support this hypothesis. Local injections of glutamate antagonists into the MLR block RS cells responses to olfactory nerve stimulation (Figure 7 in Derjean et al., 2010). In addition to driving the MLR via a glutamatergic projection, the PT also modulates its activity through a dopaminergic projection. It was shown recently that stimulation of the PT induces a dopamine release in the MLR, which increases the locomotor output by a D1 receptor-mediated mechanism (Ryczko et al., 2013). This dopaminergic drive seems to build on the glutamatergic drive to amplify the overall PT input onto the MLR. It remains to be shown, however, whether PT dopaminergic neurons are actually recruited by olfactory inputs from medial OB projection neurons. In turn, the MLR activates RS cells (Brocard and Dubuc, 2003; Le Ray et al., 2003; Brocard et al., 2010). As such, the MLR plays a key central role in initiating and controlling locomotion (Sirota et al., 2000; Le Ray et al., 2011; Ryczko and Dubuc, 2013) induced by olfactory inputs and finely tunes the power of the locomotor output.

## Comparison of the Two Sensorimotor Systems

The activation of locomotion by the two sensory modalities, cutaneous mechanoreception and olfaction, relies on the activation of RS cells in the brainstem. Cutaneous inputs activate RS cells through relay cells located in the lateral part of the hindbrain or in the dorsal column nuclei. Olfactory inputs activate RS cells through the MLR. It is well documented that the MLR activates RS cells in a graded fashion and this results in a graded locomotor output. On the other hand, cutaneous inputs generate sustained depolarizations in RS cells in an all-or-none fashion. The sustained depolarizations were recently shown to rely on intrinsic properties (I_CAN_) as well as glutamatergic synaptic transmission (Viana Di Prisco et al., 2000). Ca^2+^ imaging experiments indicate that the large RS cells are activated by both olfactory and cutaneous inputs (Viana Di Prisco et al., 1997, 2000; Derjean et al., 2010), indicating that these RS cells play a crucial role in activating locomotion induced by either sensory modality. The mechanism by which the same RS cell population could elicit graded vs. all-or-none locomotor responses have not been elucidated. It is tempting to propose that intrinsic plateau properties could be inhibited by the MLR, which is known to excite RS cells via two neurotransmitters, glutamate and acetylcholine (Le Ray et al., 2003). For instance, cholinergic inputs could suppress NMDA-induced sustained depolarizations in RS cells. There are many other possible mechanisms. The level of excitation from the MLR could be insufficient to activate the intrinsic plateau properties in RS cells, as shown in the cat preparation for sensory inputs (Brownstone et al., 1994). Another possibility would be that the intrinsic plateau properties are activated by an unidentified synaptic input originating from the cutaneous sensory inputs and not the MLR. Further work is needed to decipher between these different options.

## 5-HT Modulation

**Figure 2:**
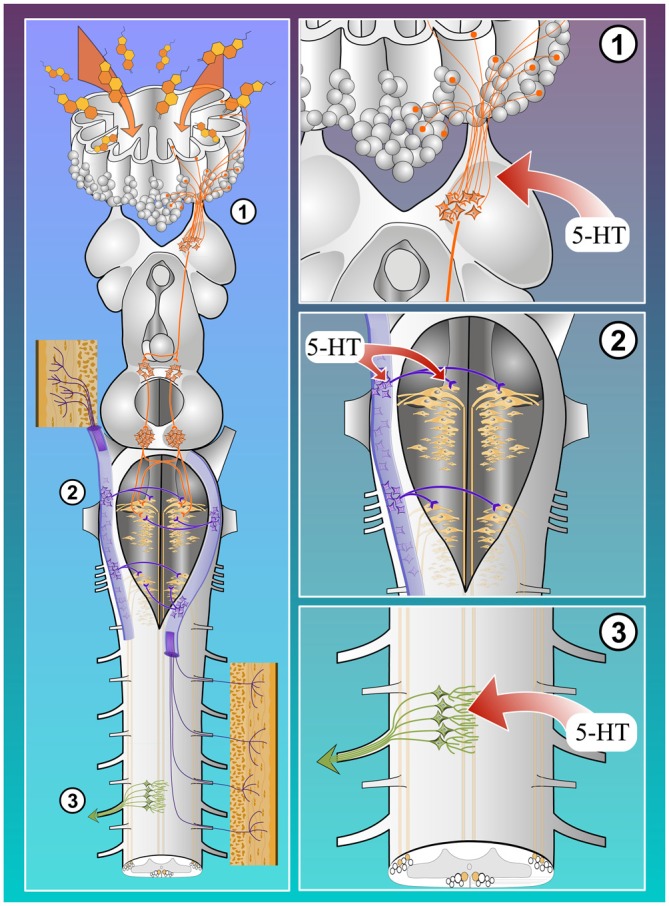
**5-HT modulation of the sensorimotor circuitry.** Both olfactory **(1)** and cutaneous **(2)** inputs to lamprey RS cells seem to be modulated by 5-HT (red arrows). 5-HT also acts at the spinal level by modulating the transmission from descending RS axons on spinal neurons **(3)**.

The general organization of the serotoninergic system in lampreys is relatively well described (Pierre et al., 1992; Antri et al., 2006; Barreiro-Iglesias et al., 2009) and it is similar to that of mammals. 5-HT modulates sensory transmission at different levels in the nervous system (Figure 2, left). For instance, several studies have shown that 5-HT modulates sensory transmission in the SC. In mammals, sensory transmission to superficial and deep dorsal horn neurons is either depressed by 5-HT (cat: Headley et al., 1978; Anwyl, 1990; and rat: Lopez-Garcia and King, 1996; Lopez-Garcia, 1998; Garraway and Hochman, 2001) or, in a small proportion of cases, potentiated by 5-HT (rat: El-Yassir et al., 1988; Lopez-Garcia and King, 1996). In tadpoles (Sillar and Simmers, 1994) and lampreys, 5-HT decreases the amplitude of EPSPs recorded in large secondary sensory neurons (giant interneurons) in response to stimulation of primary afferents (El Manira et al., 1997). In frog motoneurons, 5-HT also depresses the EPSPs induced by DR stimulation (Ovsepian and Vesselkin, 2006). Less is known about 5-HT effects on sensory transmission at the supraspinal level. A study in guinea pigs suggested that 5-HT depresses glutamate release from trigeminal primary afferents through presynaptic inhibition (Travagli and Williams, 1996). In the lamprey, 5-HT modulation occurs at several locations along the sensorimotor pathways.

### Serotoninergic Modulation of Cutaneous Inputs

Modulatory effects of 5-HT were also investigated on the transmission of sensory inputs in the brainstem of lampreys (Figure 2-2). The lamprey brainstem contains rich 5-HT innervation (Steinbusch et al., 1981). There are 5-HT fibers surrounding the cell bodies of some of the large RS cells (Viana Di Prisco et al., 1994). Moreover, there is abundant 5-HT innervation within the trigeminal descending tract (Pierre et al., 1992), where the trigeminal sensory relay cells are located (Viana Di Prisco et al., 2005). 5-HT modulation of trigeminal inputs to RS cells was investigated in the brainstem of lampreys (Antri et al., 2008). Bath application of 5-HT reduced disynaptic excitatory responses in RS cells elicited by trigeminal nerve stimulation. Similar effects were seen by local ejection of 5-HT either onto the RS cells or onto the relay cells in the lateral part of the brainstem (Figure 2-2). 5-HT also reduced the monosynaptic EPSPs elicited from stimulation of the relay cells that receive trigeminal inputs and project onto RS cells. 5-HT increased the threshold for eliciting sustained depolarizations in response to trigeminal nerve stimulation but did not prevent them. The 5-HT innervation on RS cells appeared to originate from 5-HT neurons in the isthmic region, and also from neurons located in the pretectum and caudal rhombencephalon (Figure 2-2). These results indicate that 5-HT strongly modulates sensory transmission to neural networks involved in the control of movements.

### Serotoninergic Modulation of Olfactory Inputs

In the lamprey olfactory system, 5-HT fibers were particularly prominent in the lamina propria of the olfactory epithelium, as well as in the olfactory nerve and bulb (Figure 2-1; Zielinski et al., 2000; Frontini et al., 2003; Abalo et al., 2007). On the other hand, 5-HT cell bodies were restricted to the lamina propria underlying the olfactory epithelium (Figures 1–3 in Zielinski et al., 2000). The 5-HT fibers were prominent within the olfactory nerve, parallel to the axons of the OSNs, from the lamina propria to the OB. However, some 5-HT fibers were also seen adjacent to the olfactory nerve. Cross sections of the olfactory nerve revealed that the 5-HT fibers where distributed evenly among the primary olfactory afferent fibers forming the nerve. Analysis of the pathway of individual 5-HT fibers using confocal z-series showed that these fibers terminated either at the junction of the olfactory nerve and OB or in the outer OB layers (i.e., olfactory nerve layer and glomerular layer). Olfactory nerve lesions experiments showed that these 5-HT fibers originate from cell bodies located in the mucosa of the olfactory organ (Zielinski et al., 2000). On the other hand, the abundant 5-HT innervation observed in the OB inner layers (i.e., granular) was not altered after cutting the olfactory nerve, demonstrating that this innervation has a different, probably central, origin. In lampreys, 5-HT neurons are present from the diencephalon to the caudal rhombencephalon (Antri et al., 2006). The telencephalon is devoid of serotonergic cell bodies (Pierre et al., 1992). Most afferents to the OB come from the telencephalon. However, some neurons projecting to the OB are located in the diencephalon (preoptic area) and midbrain tegmentum (Northcutt and Puzdrowski, 1988). Both these regions contain 5-HT neurons (Pierre et al., 1992; Antri et al., 2006), making them prime candidates as the central source of the OB 5-HT innervation. Interestingly, the meso-rhombencephalic group of 5-HT neurons seems to be homologous to the superior raphe of mammals (Antri et al., 2006), which is known to project to the OB and gate the olfactory information flow (Petzold et al., 2009). The function of the 5-HT innervation of the lamprey olfactory system is not fully understood (Zielinski et al., 2000). However, based on ongoing work in our group (Boyes et al., 2014), on the location of the 5-HT fibers in the OB (Zielinski et al., 2000), on how 5-HT acts on other sensory systems (Antri et al., 2008), and on the role of 5-HT in olfactory processing in other vertebrates (Petzold et al., 2009), it probably acts on olfactory processes by gating the sensory inflow.

### Serotoninergic Modulation of the Spinal Motor System

Several endogenous neurotransmitters have been shown to alter the output of locomotor CPGs and to modulate cellular and synaptic properties of the neurons involved (see for instance: Barbeau and Rossignol, 1991; Schotland et al., 1996; MacLean et al., 1998; Parker and Grillner, 2000; Schmidt and Jordan, 2000; MacLean and Schmidt, 2001; Grillner and Wallén, 2002; Alford et al., 2003; Perrier et al., 2003; Svensson et al., 2003). Little is known about the mechanisms involved. However, the subject is broad and we will not review the different neurotransmitter systems in different vertebrate species.

In lampreys, 5-HT modulates the descending motor commands in the lamprey SC in addition to gating sensory inputs to RS cells. Paracrine release of 5-HT activates at least two distinct receptor subtypes at three distinct subcellular locations with transduction mechanisms converging on a single behavioral modification. It has been observed a while ago that the frequency of fictive locomotion is modulated by endogenous release of neurotransmitters within the SC (Harris-Warrick and Cohen, 1985; Christenson et al., 1989; Schotland et al., 1996; Parker and Grillner, 2000; Svensson et al., 2003). Of these modulatory neurotransmitters, 5-HT reduces the frequency of VR bursting during fictive locomotion (Harris-Warrick and Cohen, 1985). This modulation occurs if 5-HT is applied exogenously, but it is also clear that activity-dependent release of 5-HT from within the SC occurs and that this release similarly reduces the frequency of the CPG output (Christenson et al., 1989). This behavioral outcome of 5-HT is due, in part, to 5-HT-mediated inhibition of a postsynaptic Ca^2+^-dependant K^+^-current (KCa2) that underlies the late after-hyperpolorization of action potentials in neurons of the CPG (Wallén et al., 1989a; El Manira et al., 1994; Wikström et al., 1995; Parker and Grillner, 2000). Separately and associated directly with the synaptic activation of NMDA receptors, 5-HT mediated inhibition of a postsynaptic KCa2 is thought to play a role in prolonging fictive locomotion bursts through prolonging the plateau of NMDA tetrodotoxin (TTX) oscillations (Wallén and Grillner, 1987; Christenson et al., 1989; Wallén et al., 1989b; Schotland and Grillner, 1993; El Manira et al., 1994; Alpert and Alford, 2013; Nanou et al., 2013). Finally, presynaptic 5-HT receptor activation filters synaptic output from both descending RS command neurons (Buchanan and Grillner, 1991; Blackmer et al., 2001) and from intraspinal excitatory interneurons (Parker and Grillner, 1999; Schwartz et al., 2005). This form of presynaptic inhibition causes an augmenting synaptic response that is initially inhibited but enhanced during bursting behavior.

#### Serotonin Modulation of Ca^2+^ Dependent K^+^ Conductances (KCa2) in Spinal Neurons

**Figure 3:**
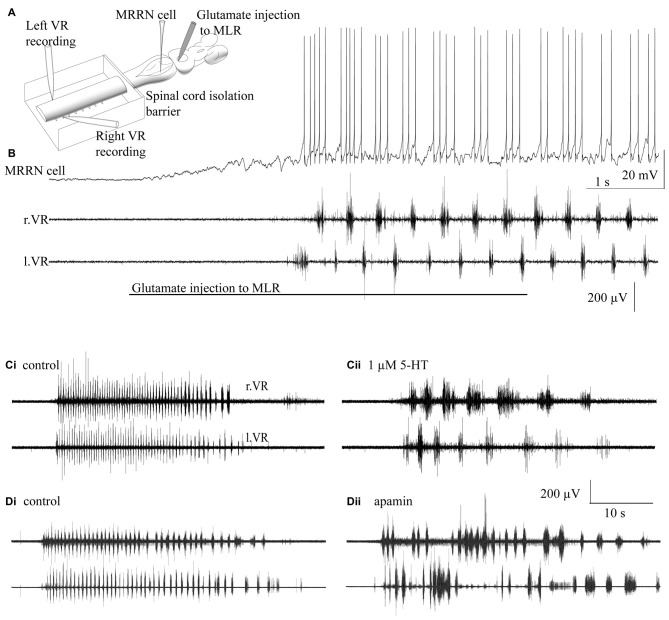
**5-HT and apamin reduce the cycle frequency of brainstem-evoked locomotion. (A)** Recording arrangement to evaluate effects of spinal agonist/antagonist application on brainstem evoked fictive locomotion. The SC was pharmacologically isolated from the brainstem with a barrier to superfusate flow at the 2nd to 5th spinal segment. Glutamate (1 mM) was microinjected into the MLR. MRRN neurons were recorded intracellularly and fictive locomotion with suction electrodes over left and right pairs of spinal VRs (l.VR, r.VR). **(B)** Injection of glutamate into the MLR evokes depolarization and rhythmic firing of the MRRN neuron and alternating VR activity. **(Ci)** Similar fictive locomotion to **(B)** recorded in a pair of VRs. **(Cii)** 5-HT (1 μM) superfused over the SC reduced fictive locomotion cycle frequencies. **(D)** Apamin—a blocker of KCa2 channels also slows MLR evoked fictive locomotion. **(Di)** Similar bout of locomotion to **(B)**. **(Dii)** Apamin (5 μM) superfused over the SC reduced the fictive locomotion frequencies. Adapted from Gerachshenko et al. (2009) and Nanou et al. (2013).

5-HT acts on most if not all neurons of the spinal CPG to inhibit the latter after hyperpolarization following action potentials (Wallén et al., 1989a). This effect may be mediated by 5-HT1A receptors to inhibit voltage gated Ca^2+^ channels (Hill et al., 2003). The consequent reduction in Ca^2+^ will inhibit the KCa2 channel activation that mediates the late after-hyperpolarization in these neurons (Wikström and El Manira, 1998). The late after-hyperpolarization strongly impacts the ability of spinal neurons to fire repetitively during bursting activity that some neurons of the CPG show and thus, 5-HT modulation of KCa2 channels is important in controlling the burst output of the CPG (Wallén et al., 1989a; Hill et al., 1992; Meer and Buchanan, 1992). Indeed, computer simulations of the lamprey CPG network that incorporate its connectivity and ionic intrinsic properties provide evidence that inhibition of KCa2 in neurons within the lamprey CPG prolongs fictive locomotion VR bursting (Hellgren et al., 1992; Lansner and Ekeberg, 1994; Grillner et al., 1998a). 5-HT also causes a prolongation of the depolarization recorded during NMDA-TTX driven intrinsic oscillations (Wallén et al., 1989a). These oscillations are mediated by intrinsic membrane properties of spinal neurons seen following NMDA receptor activation. The oscillations require Ca^2+^ permeation of the NMDA receptors and subsequent activation of a KCa2 channel. The prolongation of the depolarizing phase of these oscillations caused by 5-HT may be mediated by direct interaction of 5-HT receptors on KCa2 channels, or alternatively, via an indirect inhibition of N-methyl-D-aspartate receptors (NMDARs; Schotland and Grillner, 1993) or voltage-gated calcium channels (VGCCs; Wang et al., 2014) supplying Ca^2+^ for KCa2 channels responsible for the repolarization. More recent work ties the activation of NMDA receptors and Ca^2+^ permeation of these receptors directly to the activation of KCa2 channels, which are held in very close proximity (Alpert and Alford, 2013; Nanou et al., 2013). There is evidence that the effect of 5-HT on the NMDA mediated TTX resistant oscillations is also very important for the modulatory effects of 5-HT on the locomotor pattern. The effects of 5-HT are absent when the network is activated by kainate, which will not activate NMDARs directly. Thus, NMDAR-dependent Ca^2+^ entry contributes to burst termination (Alpert and Alford, 2013; Nanou et al., 2013). This effect is mediated by KCa2 activation, which is modified by 5-HT. These effects of 5-HT mediated through KCa2 have been shown principally in the isolated SC during fictive locomotion activated by the artificial application of NMDA. However, the effects can be readily reproduced in the SC following brainstem activation of fictive locomotion (Figure 3; Gerachshenko et al., 2009; Nanou et al., 2013).

#### Serotonin Modulates Glutamate Release in the Spinal Cord

In addition to activating a postsynaptic IK(Ca), 5-HT presynaptically inhibits synaptic transmission in the lamprey SC (Figure 2-3; Buchanan and Grillner, 1991; El Manira et al., 1994; Shupliakov et al., 1995; Blackmer et al., 2001; Takahashi et al., 2001). The inhibition of synaptic transmission by 5-HT has been observed in the CPGs of several jawed vertebrate as well. 5-HT presynaptically inhibits midcycle glycinergic inputs and prolongs VR bursting during *Xenopus* larval swimming (Sillar et al., 1998). In neonatal rat, activation of 5-HT receptors presynaptically decreases inspiratory modulated synaptic currents (Lindsay and Feldman, 1993; Di Pasquale et al., 1997; Hilaire et al., 1997) and suppresses descending glutamatergic responses (Skagerberg and Björklund, 1985). In mammalian locomotor descending command systems, 5-HT is a critical neurotransmitter. Bath applied 5-HT activates spinal rhythmic activity in rats (Cazalets et al., 1992) and refines locomotor-like activity (Pearlstein et al., 2005). 5-HT acting at 5-HT2A and 5-HT7 receptors to mediate excitatory effects (Liu and Jordan, 2005; Sławińska et al., 2014). However, 5-HT can also mediate inhibitory effects on spinal locomotor circuitry. Within descending locomotor command systems serotonin inhibits locomotor activity acting through either 5-HT1A or 5-HT1B/D receptor subtypes (Beato and Nistri, 1998; Dunbar et al., 2010), though the cellular and molecular sites of action remain unexplored. In the lamprey, the effect of presynaptic modulation of glutamatergic transmission converges on the same behavioral outcome (shown in Figure 3). This is true whether the CPG is activated by brainstem stimulation (Gerachshenko et al., 2009) or by bath application of glutamatergic agonists to the SC (Schwartz et al., 2005). The mechanism by which presynaptic 5-HT receptors mediate presynaptic modulation provides an explanation of how this convergence can occur.

In lamprey RS axons, 5-HT acting at a 5-HT1B receptor (Schwartz et al., 2005) liberates presynaptic Gβγ from the G protein heterodimer, to compete with Ca^2+^-dependent binding of the Ca^2+^ sensor for synaptic vesicle fusion, synaptotagmin, to the machinery for synaptic vesicle fusion—the soluble N-ethylmaleimide-sensitive factor attachment protein receptor (SNARE) complex (Blackmer et al., 2001, 2005; Takahashi et al., 2001; Gerachshenko et al., 2005, 2009). This competition is mediated principally by a small number of synaptosomal-associated protein 25 (SNAP-25) residues (Wells et al., 2012). Rather than causing a reduction in the probability of release at these synapses, this competitive interaction between Gβγ and synaptotagmin reduces synaptic cleft glutamate concentrations (Schwartz et al., 2007), by causing kiss-and-run fusion of the synaptic vesicles (Photowala et al., 2006). Lower synaptic glutamate concentrations cause AMPA receptor excitatory post synaptic currents (EPSCs) to be more profoundly inhibited than NMDA receptor EPSCs (Schwartz et al., 2007). Thus, 5-HT causes a differential inhibition with much stronger inhibition of AMPA mediated EPSCs. This effect is seen both at RS synapses and in synapses from intraspinal excitatory interneurons (Schwartz et al., 2005; Gerachshenko et al., 2009). Interestingly, fictive locomotion mediated by NMDA receptor activation is much slower than that mediated by AMPA or kainate application (Brodin et al., 1985; Brodin and Grillner, 1986). This is one means by which presynaptic 5-HT receptors slow fictive locomotion. However, the effect of Gβγ on modulating neurotransmitter release is dependent upon its competition with synaptotagmin in binding to the SNARE complex. Synaptotagmin binding to the SNARE complex is Ca^2+^-dependent. Consequently, at high Ca^2+^ concentrations, preferential synaptotagmin binding to SNARE complexes occludes 5-HT receptor-mediated inhibition (Yoon et al., 2007) by displacing Gβγ from the SNARE complex. During bursting activity, Ca^2+^ concentrations in the presynaptic terminals summate. This rising Ca^2+^ concentration inactivates 5-HT mediated presynaptic inhibition within 3–5 action potentials of a 50 Hz burst (Gerachshenko et al., 2009). Computational simulations of the spinal circuitry for locomotion reveal that such an augmenting excitatory synaptic signal within the spinal CPG mediates a slower fictive locomotor activity (Hellgren et al., 1992; Parker and Grillner, 1999). In mammalian systems, the cellular mechanisms of action of 5-HT are less well understood, but a similarly complex series of effects has been associated with excitatory 5-HT2 and 5-HT7 receptors and inhibitory 5-HT1 receptors. Thus, 5-HT release within the mammalian SC both facilitates locomotion but also modulates rhythmic activity (Perrier and Cotel, 2015).

#### Summary of the Effects of 5-HT on the Spinal CPG

5-HT can be released within the SC (Figure 2-3), and its release causes a slowing of fictive locomotion, whether activated by exogenous agonists, or by brainstem stimulation. However, at least three functionally distinct receptors mediate effects that converge on this behavioral outcome. Two postsynaptic receptors cause a reduction in the activation of a KCa2 channel. One of these inhibits the action potential late after-hyperpolarization to sustain spiking during bursts, the other prevents burst termination by inhibiting an NMDA receptor dependent activation of a KCa2. Presynaptically, 5-HT1B receptors inhibit release by causing kiss and run fusion. This effect favors activation of postsynaptic NMDA receptors over AMPA, but also the inhibition is lost during bursts. Both of these presynaptic effects contribute to a slowing of the CPG frequency. Further studies are needed to establish whether similar mechanisms act at the sensory level and how 5-HT modulation in the SC impacts sensory-evoked locomotion.

## General Conclusions

Sensory inputs from different modalities induce locomotion. Studies in lampreys have been extremely useful for gaining an understanding of the function and the regulation of these mechanisms. Both cutaneous and olfactory inputs impinge on RS cells that constitute the final common descending pathway for eliciting locomotion. Yet, the mechanisms by which RS cells are activated differ according to the sensory modality. Future research should indicate more precisely how graded vs. all-or-none locomotor output is elicited by olfactory vs. cutaneous inputs. Moreover, modulatory mechanisms also play a crucial role in gating the sensory inflow and determining the strength of the locomotor output. For instance, we have discussed how 5-HT acts at all levels of the sensorimotor pathways via different modulatory mechanisms.

## Author Contributions

GD, WWG, STA, BSZ, and RD wrote the article.

## Funding

We acknowledge the support of the Great Lakes Fishery Commission over the years, as group grants to BZ and RD (GLFC, 54011, 54021, 54035). Group Grants provide the necessary conditions to promote in-depth reviews of this kind. This work was also supported by a grant to RD from the Natural Sciences and Engineering Research Council of Canada (NSERC, 217435), a grant from the Canadian Institutes of Health Research (CIHR, 15129) and a group grant from the Fonds de Recherche du Québec - Santé (FRQS, 5249).

## Conflict of Interest Statement

The authors declare that the research was conducted in the absence of any commercial or financial relationships that could be construed as a potential conflict of interest.

## Abbreviations

5-HT: 5-hydroxytryptamine
AMPA: α-amino-3-hydroxy-5-methyl-4-isoxazolepropionic acid
AOO: accessory olfactory organ
ARRN: anterior rhombencephalic reticular nucleus
CPG: central pattern generator
DR: dorsal root
dV: descending root of the trigeminal nerve
DCN: dorsal column nucleus
EPSC: excitatory post synaptic current
EPSP: excitatory post synaptic potential
MLR: mesencephalic locomotor region
MOE: main olfactory epithelium
MRN: mesencephalic reticular nucleus
MRRN: middle rhombencephalic reticular nucleus
ndV: nucleus of the descending root of the trigeminal nerve
NMDA: N-methyl-D-aspartate
NMDAR: N-methyl-D-aspartate receptor
OB: olfactory bulb
OLA: octavolateralis area
OSN: olfactory sensory neurons
PRRN: posterior rhombencephalic reticular nucleus
PT: posterior tuberculum
RS: reticulospinal
SC: spinal cord
SNAP-25: synaptosomal-associated protein 25
SNARE: soluble N-ethylmaleimide-sensitive factor attachment protein receptor
TTX: tetrodotoxin
VGCC: voltage-gated calcium channel
VR: ventral root.
